# Establishment of a risk prediction model for residual back pain after percutaneous kyphoplasty in osteoporotic vertebral compression fractures

**DOI:** 10.3389/fsurg.2025.1625518

**Published:** 2025-08-13

**Authors:** Yi Rong, Yihua Zhu, Heng Yin, Hao Yu, Jianwei Wang, Yang Shao, Shaoshuo Li, Jiapeng Ye, Yang Guo, Yong Ma, Lining Wang, Zhen Hua

**Affiliations:** ^1^ Department of Traumatology & Orthopedics, Wuxi Affiliated Hospital of Nanjing University of Chinese Medicine, Wuxi, China; ^2^ Laboratory of New Techniques of Restoration & Reconstruction, Institute of Traumatology & Orthopedics, Nanjing University of Chinese Medicine, Nanjing, China; ^3^ Jiangsu CM Clinical Innovation Center of Degenerative Bone & Joint Disease, Wuxi, China; ^4^ School of Integrated Chinese and Western Medicine, Nanjing University of Chinese Medicine, Nanjing, China; ^5^ Department of Orthopedic Surgery, Nanjing Hospital of Chinese Medicine Affiliated to Nanjing University of Chinese Medicine, Nanjing, China

**Keywords:** osteoporotic vertebral compression fracture, percutaneous kyphoplasty, residual back pain, regression analysis, risk prediction

## Abstract

**Purpose:**

Severe residual back pain (RBP) after percutaneous kyphoplasty (PKP) significantly impacts postoperative prognosis and quality of life in patients. The aim of this study was to identify the risk factors for RBP in osteoporotic vertebral compression fracture (OVCF) patients after PKP, to establish a risk prediction model, and to validate its effectiveness.

**Methods:**

A case-control study was carried out among OVCF patients, who were assigned to either the training set (these patients were recruited from January 2018 and June 2020) or the validation set (these patients were recruited from July 2020 and December 2020). Risk factors were identified by univariate analysis and multifactor logistic regression analysis. The performance of the prediction model was determined by using the area under the receiver operating characteristic (ROC) curve (AUC) to assess discrimination. A nomogram for risk prediction was constructed, the Hosmer-Lemeshow test and calibration curves were used to assess calibration, and decision curve analysis was used to assess the clinical use of the model.

**Results:**

A total of 647 patients were included, 569 cases were used to train the model and 78 cases were used for external validation. Based on the data of model training set, age, bone mineral density, trauma history, posterior fascial edema, platelet distribution width, serum chloride, and middle vertebral height were independent risk factors for RBP after PKP (*P* ≤ 0.05). The AUC of the risk prediction model constructed thus was 0.788 (95% CI, 0.740–0.836), cut off (0.710, 0.761), with good discrimination. Calibration curves of the model training and validation sets were between the standard curve and the acceptable line, and the Hosmer-Lemeshow test indicated that the model training and validation sets were *χ*^2^ = 6.354 and *χ*^2^ = 7.240, (*P* = 0.608 and 0.511), respectively, which have good calibration. The decision curve analysis showed that the threshold probability interval of the net benefit value of the model was 6.3%–82.3% for the training set, 8.7%–55.6% and 72.5%–81.3% for the validation set.

**Conclusion:**

The constructed model showed good predictive ability in the occurrence of residual back pain after PKP, which can provide a scientific basis and guidance for clinical prevention and treatment.

## 1 Introduction

As society ages, the incidence of osteoporosis is increasing, with decreased bone strength increasing susceptibility to fracture (1). In 2000, there were 1.4 million vertebral fractures worldwide, however, it is projected that the number of cases will reach 3 million by 2050 in China alone (2). It usually lacks a clear trauma history or occurs after low-energy injury neglected by patients, resulting in persistent severe pain, local vertebral kyphosis, respiratory dysfunction, increased risk and mortality of new fractures, and severe decline in quality of life (3). Currently, the clinical treatment of OVCF is categorized into conservative and surgical treatments (4). Conservative treatment includes bed rest, or wearing a brace, along with medication such as painkillers and muscle relaxants. In contrast, minimally invasive surgery is typically used for surgical treatment, including percutaneous vertebroplasty (PVP) and percutaneous kyphoplasty (PKP). By injecting polymethylmethacrylate (PMMA) bone cement, vertebral bone strength can be increased and mechanical stability can be achieved by increasing the space between vertebrae, thus providing immediate pain relief (5). Compared to conservative treatment, minimally invasive surgery has advantages in improving patients' quality of life and prolonging survival time (6). Nonetheless, some patients still suffer from residual back pain after PVP and PKP, which hinders early ambulation and functional recovery due to moderate or severe pain (7, 8).

Currently, the risk factors for RBP after PVP have been well-studied, which include low bone mineral density (BMD), presence of intravertebral cleft, paravertebral muscle degeneration, sarcopenia, multilevel OVCFs, unsatisfactory bone cement distribution, insufficient bone cement filling, unrecoverable vertebral height, large pelvic angle of C7 vertical sagittal axis (SVA) T1 (TPA), and lumbar lordosis (LL)-pelvic incidence (PI) mismatch, etc (9–12). However, few studies have described risk factors for RBP after PKP. In the clinical setting, many patients have high expectations for their prognosis after minimally invasive surgery. Once RBP occurs in the postoperative period, patient dissatisfaction or hostility can easily be aroused. Meanwhile, there is a lack of early and accurate prediction and diagnosis of postoperative RBP, relying mainly on patient self-reporting during postoperative rounds. Therefore, it is crucial to identify risk factors for postoperative RBP in patients with OVCF. On the one hand, it allows for early intervention in patients with RBP to improve prognosis. On the other hand, relatively accurate prediction enables clinicians to identify high-risk patients and control their expectations. In this study, we hope to establish a nomogram model that can predict residual back pain after PKP by comprehensively analyzing the risk factors, the diagnostic effects of the independent risk factors, and their respective and comorbid factors, so as to provide a basis and guidance for effective clinical prevention and treatment.

## 2 Materials and methods

### 2.1 Participants

A total of 647 patients with OVCF who received treatment in the orthopedic and traumatology ward of the Wuxi Traditional Chinese Medicine Hospital were selected from January 2018 to December 2020. Data from 569 patients between January 2018 and June 2020 were used as the model training set, including 122 males and 447 females aged 50–97 years (mean ± SD, 71.76 ± 9.066 years). Data from 78 patients collected between July 2020 and December 2020 were used as the model validation set, including 16 males and 62 females, aged 51–96 years (mean ± SD, 73.04 ± 10.43 years). This study was conducted in accordance with the principles outlined in the Declaration of Helsinki and approved by the Institutional Review Board of Wuxi Hospital of Traditional Chinese Medicine (Approval Number: SSF2022022504). All patients provided signed informed consent.

The diagnostic criteria for osteoporotic vertebral compression fractures were (13): (1) Having a history of osteoporotic fracture or slight trauma, persistent chest, waist, and back pain; pain relief or disappearance when lying down and resting; and pain worsening when changing posture; physical examination indicating activity of the chest and waist was limited, and the vertebrae involved in the fracture showed tenderness and percussion pain. Generally, there is no evidence of lower limb nerve damage, the height was short, or the back was deformed. (2) Imaging examination: radiographic imaging examination revealed a wedge-shaped change or “double concave sign”, and some showed a “vacuum sign” in the vertebral body and the formation of false joints. Magnetic resonance imaging (MRI) revealed a hypointense fracture on T1WI, hyperintense or isointense signs on T2WI, and hyperintense signs on the lipid suppression sequence. (3) BMD examination: Dual-energy x-ray absorptiometry (DXA) was used to determine the *T* value at the spine/hip joint ≤−2.5.

Diagnostic criteria for RBP (11): VAS scores ≥4 at 3 days and 1 month after the operation. The patients were divided into a pain group (*n* = 117) and a no pain group (*n* = 452) based on the diagnostic criteria of postoperative RBP.

The inclusion criteria in our study were: (1) meets the diagnostic criteria of OVCF and has no neurological injury; (2) received PKP surgery; (3) a single vertebra was responsible for the fracture; (4) meets the diagnostic criteria of RBP; (5) Clinical records and follow-up data were complete.

The exclusion criteria in our study were: (1) previous spinal surgery; (2) Previous chronic low back pain history, such as fasciitis, postherpetic neuralgia, etc. (3) OVCF patients caused by tumor, infection, or tuberculosis; (4) patients with coagulation dysfunction or systemic disease who cannot tolerate surgery; (5) systemic or local infection; (6) new vertebral fracture occurred after the operation; (7) spinal cord compression and obvious neurological symptoms, such as numbness and/or muscle weakness; (8) clinical medical records and follow-up data were incomplete.

### 2.2 Surgical method and postoperative management

Patients were placed in the prone position, the chest and hip were positioned on a pillow, and the waist was extended. Under the guidance of C-arm fluoroscopy, the pedicle shadow was located on both sides of the fractured vertebral body. Following 1% lidocaine anesthesia, a small opening was cut. The cook needle was inserted through the pedicle of both sides of the vertebral fracture body to 0.5–0.8 cm from the posterior edge of the vertebral body, the guide needle was inserted, and the cook needle was retrieved and then inserted into the working channel. The guide pin was removed, the cancellous bone in the vertebral body was placed 0.5 cm from the leading edge of the vertebral body with the core of the bone cement pusher, and the expansion balloon was expanded to 2.0 ml. The bone cement was prepared and injected into the vertebral body from the working channels on both sides of the patient by the bone cement pusher during the agglomeration period. The bone cement was filled, and the patient reported no adverse reactions. The working channel was removed, and pressure was applied to stop the bleeding. All patients received vitamin D and salmon calcitonin postoperatively. The patients were examined by anteroposterior and lateral x-rays 24 h postoperatively and were discharged 2–3 days after the operation. Radiographs of the injured vertebrae were regularly examined postoperatively. Patients did not receive non-steroidal anti-inflammatory drugs (NSAIDs) or opioid analgesics postoperatively unless the patient's postoperative pain was not relieved. Specifically, NSAIDs are preferred if the VAS score is between 4 and 6, and opioids are considered if NSAIDs remain ineffective or the pain is severe with a VAS score >6.

### 2.3 Date collection

**Figure 1:**
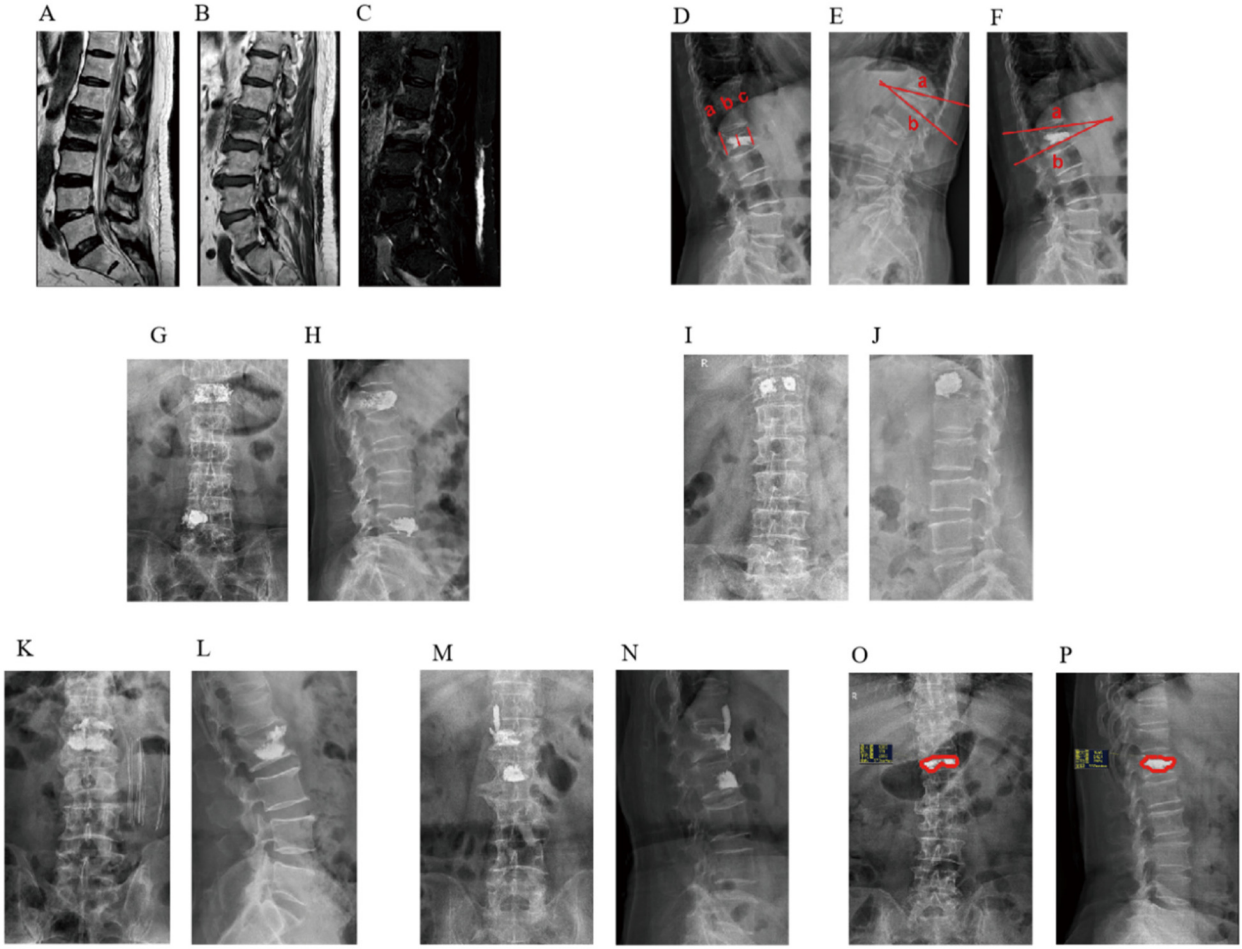
MRI findings of fascial edema after osteoporotic vertebral compression fracture. **(A)** T1WI, **(B)** T2WI, **(C)** T2-STIR WI; Imaging evaluation of the vertebral body of osteoporotic vertebral compression fracture. **(D)** Anterior vertebral height (AVH), Middle vertebral height (MVH), Postoperative Posterior vertebral height (PVH), **(E)** Preoperative Cobb angle, **(F)** Postoperative Cobb angle; Distribution characteristics of spongy bone cement. **(G)** anteroposterior radiograph of spongy diffuse distribution pattern, **(H)** lateral radiograph of spongy diffuse distribution pattern; Distribution characteristics of massive bone cement. **(I)** anteroposterior radiograph film of the local solid distribution pattern of massive bone cement, **(J)** lateral radiograph of massive local solid distribution pattern; Image of bone cement leaking into the intervertebral space. **(K)** anteroposterior radiograph of bone cement leaking into the intervertebral space, **(L)** lateral radiograph of bone cement leaking into the intervertebral space; Image of bone cement leaking into the paravertebral tissue. **(M)** anteroposterior radiograph of bone cement leaking into the paravertebral tissue, **(N)** lateral x-ray film of bone cement leaking into paravertebral tissue; Bone cement perfusion ratio. **(O)** coronal bone cement perfusion ratio, **(P)** sagittal bone cement perfusion ratio.

Relevant information regarding patients was collected by referring to electronic medical records. Preoperative, intraoperative, and postoperative factors that may affect back pain were evaluated, including (1) general data: sex, age, height, weight and body mass index (BMI); (2) preoperative complications: diabetes, hypertension, pulmonary disease, cardiovascular, and cerebrovascular disease; (3) clinical data: trauma history, fracture segment, lumbar and dorsal fascia edema, BMD, time from injury to operation and duration of operation; (4) Laboratory examination: preoperative blood cell analysis, liver and kidney function, and coagulation function; (5) Preoperative and postoperative radiological parameters: Cobb angle of the fractured vertebral body before the operation, whether the heights of the anterior, middle, and posterior vertebral bodies are restored 24 h after the operation, Cobb angle, volume, distribution and shape of bone cement infusion, leakage and position of bone cement, and recovery rate of the vertebral body (Figure 1).

### 2.4 Prediction model construction

In the model training set, the independent risk factors of RBP after PKP were analyzed by multivariate logistic regression, with the factors with significant differences screened by univariate analysis as independent variables. Based on this, the nomogram model for predicting the risk of RBP after PKP in OVCF patients was further constructed using RStudio software.

### 2.5 Model evaluation

ROC curves were used to assess the sensitivity and specificity of the model in predicting the occurrence of residual back pain after PKP. Calibration curves were drawn to verify the consistency between the predicted and the actual risk. The clinical decision curve was used to verify the clinical applicability of the model. Using Bootstrap, the training set was repeatedly sampled 1,000 times for internal validation of the model, while the validation set was used for external validation.

### 2.6 Statistical analysis

SPSS 25.0 software was used to analyze the differences between the two groups. For continuous variables that are consistent with the normal distribution, two independent sample t-tests were conducted. The rank sum test was used for continuous variables that did not conform to normal distribution. For categorical variables, the chi-square test was used for statistical analysis. *P*-values <0.05 were considered statistically significant, and *α* = 0.05 (both sides) was the inspection level. ROC curves, the nomogram model, calibration curve, and clinical decision curve was obtained using RStudio software 4.4.2.

## 3 Results

### 3.1 Univariate analysis of residual back pain after PKP in patients with OVCF

Of the 569 patients who underwent PKP surgery, 117 (20.56%) were classified as the postoperative RBP group, and 452 (79.44%) in the same period were identified as the pain-free group. Univariate analysis was performed on the clinical data of the two groups of patients with postoperative RBP. Age, sex, height, weight, BMD,trauma history, posterior fascia edema, platelet distribution width (PDW), serum chlorine (CL), lactate dehydrogenase (LDH), whether bone cement was poured to the lower edge, bone cement volume, whether or not the height of the anterior (AVH) and middle vertebrae (MVH) is restored, and recovery rate of the vertebrae was statistically significant (*P* < 0.05); There were no significant differences between the two groups in BMI, fracture segment, fracture-to-operation time, operation duration, combined medical basic diseases, other laboratory examination results and operation factors (*P* > 0.05) (Tables 1–3).

**Table 1:** Univariate analysis of clinical data of OVCF patients with residual back pain after PKP.

Factor	Total number (569 cases)	Pain-free group (452 cases)	Pain group (117 cases)	*P*-value	*T*-value
Age (years)	71.76 ± 9.066	72.23 ± 9.08	69.19 ± 8.56	0.001	3.273
Sex (%)					
Male (cases)	122 (21.44)	86 (19.03)	36 (30.77)	0.006	7.609
Female (cases)	447 (78.56)	366 (80.97)	81 (69.23)		
Height (cm)	160.03 ± 7.02	159.59 ± 6.86	161.54 ± 7.95	0.008	−2.645
Weight (kg)	59.20 ± 10.02	58.76 ± 10.04	61.33 ± 9.60	0.013	−2.488
BMI	23.05 ± 3.23	23.01 ± 3.28	23.45 ± 2.90	0.187	−1.321
BMD (%)					
|*t*| > 3.5 (cases)	251 (44.11)	217 (48.01)	34 (29.06)	0.000	13.53
2.5 ≤ |*t*| ≤ 3.5 (cases)	318 (55.89)	235 (51.99)	83 (70.94)		
Trauma (%)					
Yes (cases)	420 (73.81)	320 (70.80)	100 (85.47)	0.001	10.35
No (cases)	149 (26.19)	132 (29.20)	17 (14.53)		
Posterior fascia edema (%)					
Yes (cases)	52 (9.14)	25 (5.53)	27 (23.08)	0.000	34.45
No (cases)	517 (90.86)	427 (94.47)	90 (76.92)		
Fracture segment	–			0.636	8.842
Injury time	11.23 ± 22.40	9.33 ± 14.31	9.76 ± 13.41	0.769	−0.294
Operation duration (min)	49.97 ± 17.79	48.22 ± 16.49	48.85 ± 16.77	0.714	−0.367
Complication (%)					
Hypertension (cases)	154 (27.07)	114 (31.86)	40 (34.19)	0.052	3.786
Diabetes (cases)	44 (7.73)	38 (8.41)	6 (5.13)	0.237	1.400
Pulmonary disease (cases)	8 (1.41)	7 (1.55)	1 (0.85)	0.570	0.323
Cardiovascular disease (cases)	38 (6.68)	29 (6.42)	9 (7.69)	0.622	0.243
Cerebrovascular disease (cases)	32 (5.62)	25 (5.53)	7 (5.98)	0.850	0.036

**Table 2:** Univariate analysis of laboratory examination of patients with residual back pain after PKP in OVCF.

Factor	Pain free group (452 cases)	Pain group (117 cases)	*P*-value	*T*-value
RBC (10^12^/L)	4.23 ± 0.44	4.20 ± 0.44	0.473	0.718
WBC (10^9^/L)	6.23 ± 1.91	6.08 ± 1.75	0.434	0.783
PLT (10^9^/L)	203.27 ± 66.11	194.98 ± 64.89	0.225	1.214
HGB (g/L)	127.14 ± 13.38	126.46 ± 12.27	0.621	0.495
LYMPH%	24.55 ± 8.55	23.96 ± 8.72	0.511	0.657
NEUT%	67.10 ± 9.49	67.49 ± 9.80	0.693	−0.395
HCT%	38.80 ± 3.78	38.66 ± 3.78	0.709	0.374
MCH (pg)	30.09 ± 1.50	30.20 ± 1.59	0.512	−0.656
MCHC (g/L)	327.55 ± 10.00	327.01 ± 9.40	0.598	0.527
MCV (fL)	91.87 ± 3.93	92.34 ± 4.53	0.262	−1.123
PDW	12.69 ± 2.35	13.27 ± 2.61	0.020	−2.326
RDW-CV	13.19 ± 0.84	13.27 ± 0.82	0.376	−0.887
CRP (mg/L)	16.09 ± 20.85	20.08 ± 28.19	0.089	−1.703
K (mmol/L)	3.91 ± 0.35	3.86 ± 0.36	0.175	1.358
NA (mmol/L)	140.11 ± 2.68	139.97 ± 3.20	0.635	0.475
CL (mmol/L)	104.07 ± 3.04	103.06 ± 4.28	0.004	2.910
CA (mmol/L)	2.27 ± 0.10	2.26 ± 0.11	0.453	0.750
ALT (U/L)	17.91 ± 17.71	19.50 ± 16.84	0.385	−0.869
AST (U/L)	22.57 ± 16.91	22.70 ± 6.78	0.935	−0.082
CH (mmol/L)	4.62 ± 0.98	4.61 ± 0.98	0.906	0.118
TG (mmol/L)	1.35 ± 0.74	1.29 ± 0.54	0.374	0.890
GLU (mmol/L)	5.68 ± 1.68	5.57 ± 1.09	0.497	0.680
UREA (mmol/L)	281.81 ± 80.89	276.83 ± 80.82	0.553	0.593
CREA (umol/L)	60.51 ± 15.18	58.55 ± 13.45	0.203	1.274
CHE (U/L)	7,111.31 ± 1,625.56	6,990.16 ± 1,724.66	0.478	0.709
LDH (U/L)	203.06 ± 53.57	214.60 ± 54.24	0.039	−2.072
ALP (U/L)	104.09 ± 35.82	99.99 ± 43.37	0.293	1.053
TP (g/L)	67.32 ± 4.93	67.13 ± 5.49	0.722	0.356
CK (U/L)	90.82 ± 166.35	85.27 ± 99.32	0.730	0.345
CKMB9 (ng/ml)	11.38 ± 8.62	11.20 ± 5.80	0.836	0.207
TBIL (μmol/L)	16.01 ± 9.58	16.06 ± 6.62	0.961	−0.048
IBIL (μmol/L)	13.14 ± 6.52	13.27 ± 5.59	0.841	−0.201
DBIL (μmol/L)	2.88 ± 4.10	2.79 ± 1.26	0.818	0.230
GLD (g/L)	28.21 ± 4.26	27.92 ± 4.01	0.502	0.671
GSP (mmol/L)	230.02 ± 57.61	231.40 ± 48.95	0.812	−0.237
AMY (U/L)	56.42 ± 24.12	53.66 ± 22.57	0.265	1.115
DD2 (ng/ml)	2.44 ± 3.45	2.24 ± 2.56	0.555	0.591
FIB-RP (mg/dl)	3.37 ± 0.82	3.32 ± 0.74	0.569	0.570
APTT(s)	28.58 ± 6.21	28.72 ± 6.12	0.820	−0.228
FDP (mg/L)	7.66 ± 12.27	7.56 ± 10.78	0.940	0.075
TT(s)	17.50 ± 4.60	17.16 ± 1.96	0.441	0.771
INR	0.93 ± 0.10	0.94 ± 0.07	0.827	−0.218
PT(s)	11.54 ± 1.37	11.54 ± 1.10	0.991	0.011
Myo (μg/L)	19.19 ± 17.70	19.83 ± 25.63	0.755	−0.312
NT-proBNP (pg/ml)	448.51 ± 1,667.14	470.05 ± 733.73	0.892	−0.136
PCT (ng/ml)	0.21 ± 0.07	0.20 ± 0.06	0.469	0.724

**Table 3:** Univariate analysis of surgical factors of patients with residual back pain after PKP in OVCF.

Factor	Pain-free group (452 cases)	Pain group (117 cases)	*P*-value	T-value
Leakage of boneless cement (cases, %)	430 (95.13)	108 (92.31)	0.230	1.440
Bone cement leakage (cases, %)	22 (4.87)	9 (7.69)		
Leakage into intervertebral space (cases, %)	16 (3.54)	8 (6.84)	0.114	2.502
Leakage into paravertebral tissue (cases, %)	9 (1.99)	1 (0.85)	0.404	0.695
Poured to the upper edge (cases, %)	327 (72.35)	74 (63.25)	0.055	3.697
Poured to the lower edge (cases, %)	309 (68.36)	68 (58.12)	0.037	4.362
Massive bone cement (cases, %)	271 (59.96)	68 (58.12)	0.718	0.130
Bone cement volume (ml)	5.07 ± 1.49	4.64 ± 0.83	0.003	2.955
AVH restored (cases, %)	343 (75.88)	68 (58.12)	0.000	14.625
MVH restored (cases, %)	382 (84.51)	66 (56.41)	0.000	43.841
PVH restored (cases, %)	446 (98.67)	116 (99.15)	0.679	0.171
Preoperative cobb angle (°)	11.36 ± 5.53	10.39 ± 5.71	0.096	1.667
Postoperative cobb angle (°)	7.60 ± 4.84	7.52 ± 5.21	0.875	0.158
Coronary perfusion ratio (%)	43.78 ± 13.74	43.79 ± 13.61	0.998	−0.002
Sagittal perfusion ratio (%)	44.86 ± 12.13	44.76 ± 12.55	0.941	0.075
Recovery rate (%)	36.02 ± 24.95	30.52 ± 24.42	0.033	2.136

Recovery rate, (preoperative Cobb angle—postoperative Cobb angle)/preoperative Cobb angle).

### 3.2 Multivariate logistic regression analysis of residual back pain after PKP in patients with OVCF

Multivariate logistic regression analysis showed that age, BMD value of 2.5 |*t*| ≤ 3.5, trauma history, posterior fascial edema, platelet distribution width, serum chlorine, and middle vertebral height were independent risk factors for RBP after PKP in patients with OVCF (*P* < 0.05) (Table 4). Hosmer-Lemeshow test indicated a good fit (*x*^2^ = 6.354, *P* = 0.608, *P* > 0.05). The assignment of each factor is shown in Table 5.

**Table 4:** Multivariate logistic regression analysis of residual back pain after PKP in OVCF patients.

Risk factors	Regression coefficient	OR	*P*-value	95% confidence interval	
				Lower	Upper
Age	−0.038	0.963	0.007	0.937	0.990
Sex	0.295	1.343	0.360	0.715	2.522
Height	0.012	1.012	0.596	0.968	1.057
Weight	0.001	0.954	0.954	0.971	1.032
BMD's |*t*| > 3.5	−0.553	0.575	0.035	0.344	0.962
Trauma history	0.797	2.219	0.013	1.182	4.165
PFO	1.804	6.076	0.000	3.079	11.990
PDW	0.139	1.149	0.003	1.049	1.260
CL	−0.097	0.908	0.004	0.850	0.969
LDH	0.002	1.002	0.274	0.998	1.007
Bone cement volume	−0.194	0.824	0.103	0.652	1.040
Poured to the lower edge	−0.205	0.815	0.427	0.491	1.351
AVH restored	−0.211	0.810	0.436	0.477	1.376
MVH restored	−1.349	0.260	0.000	0.154	0.437
Recovery rate	−0.452	0.637	0.391	0.227	1.787
Constant	8.689	5,935.06	0.075		

**Table 5:** Multivariate logistic regression analysis variable assignment.

Variable	Assignment
Age	continuous variable
Sex	Female = 0, Male = 1
Height	continuous variable
Weight	continuous variable
BMD|*t*| > 3.5	No = 0, Yes = 1
Trauma history	No = 0, Yes = 1
PFO	No = 0, Yes = 1
PDW	continuous variable
CL	continuous variable
LDH	continuous variable
Bone cement volume	continuous variable
Poured to the lower edge	No = 0, Yes = 1
AVH restored	No = 0, Yes = 1
MVH restored	No = 0, Yes = 1
Recovery rate	continuous variable
RBP	No = 0, Yes = 1

### 3.3 ROC curve analysis of independent risk factors and their combined models

**Figure 2:**
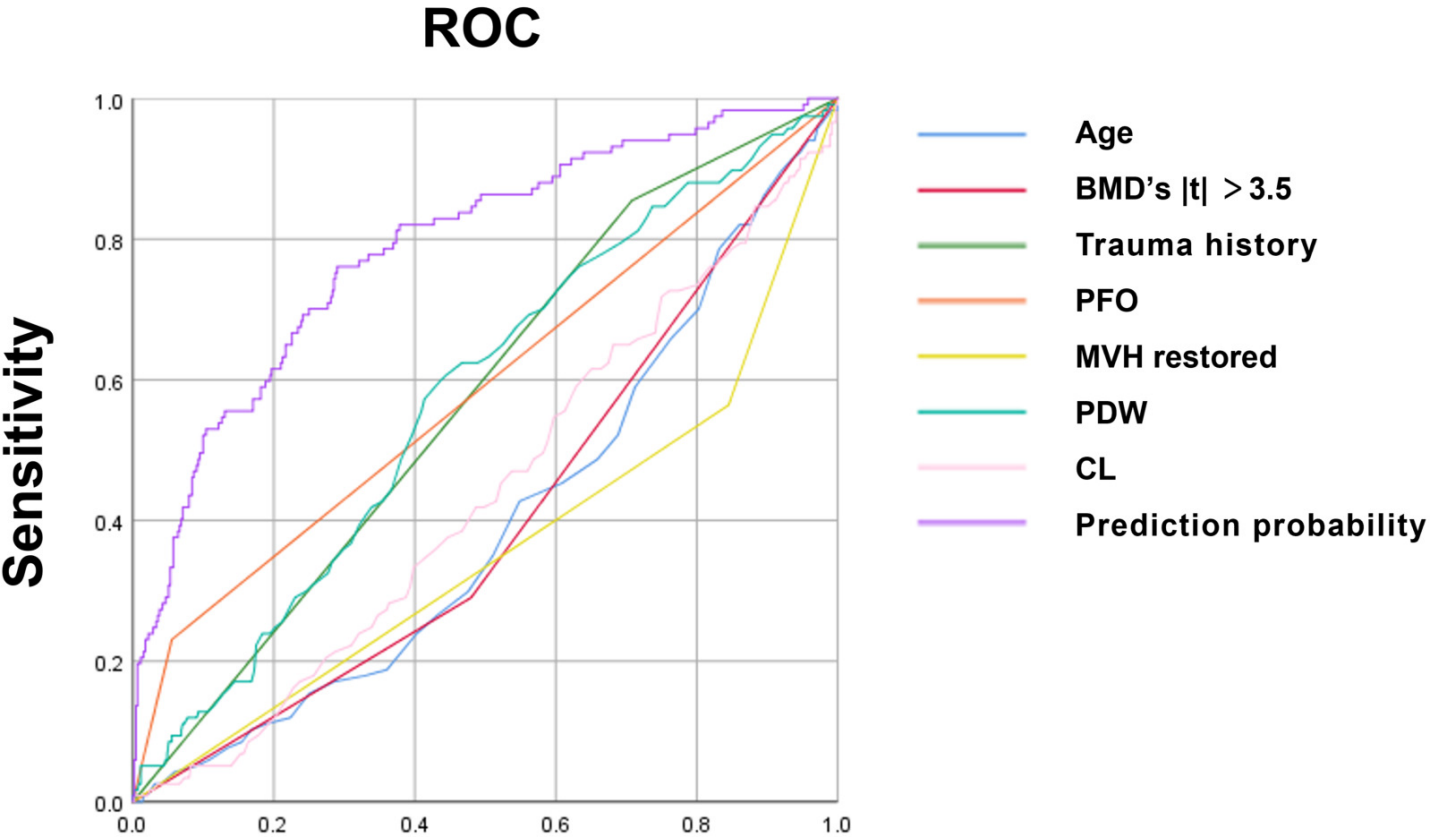
ROC curve of each independent risk factor and joint model developed using SPSS25.0 software.

The ROC curve analysis was conducted on various independent risk factors, as well as their combined models. The analysis revealed that the area under the curve (AUC) for each individual risk factor was as follows: Age (0.600), BMD (0.595), trauma history (0.573), posterior fascia edema of OVCF (PFO) (0.588), platelet distribution width (0.575), and serum chlorine (0.561). Furthermore, the height of the middle vertebral body did not recover (0.641), and the combined model had an AUC of 0.788 (95% CI, 0.740–0.836) with cut-off values of 0.710 and 0.761, respectively. All of these results were statistically significant (*P* < 0.05). The model had good discrimination and a high diagnostic value (Figure 2, Table 6).

**Table 6:** ROC curve of each independent risk factor.

Factor	AUC	*P*-value	Sensitivity %	Specificity %	95% confidence interval	
					Lower	Upper
Age	0.600	0.029	70.1	52.4	0.544	0.656
BMD's |*t*| > 3.5	0.595	0.029	70.9	52	0.539	0.651
Trauma history	0.573	0.028	85.5	70.8	0.518	0.629
PFO	0.588	0.032	23.1	5.5	0.526	0.650
PDW	0.575	0.029	60.7	44.5	0.518	0.632
CL	0.561	0.029	70.9	61.3	0.504	0.618
MVH restored	0.641	0.031	43.6	15.5	0.580	0.701
Prediction probability	0.788	0.024	76.1	29.0	0.740	0.836

### 3.4 Construction of a nomogram model to predict the risk of residual back pain after PKP in patients with OVCF

**Figure 3:**
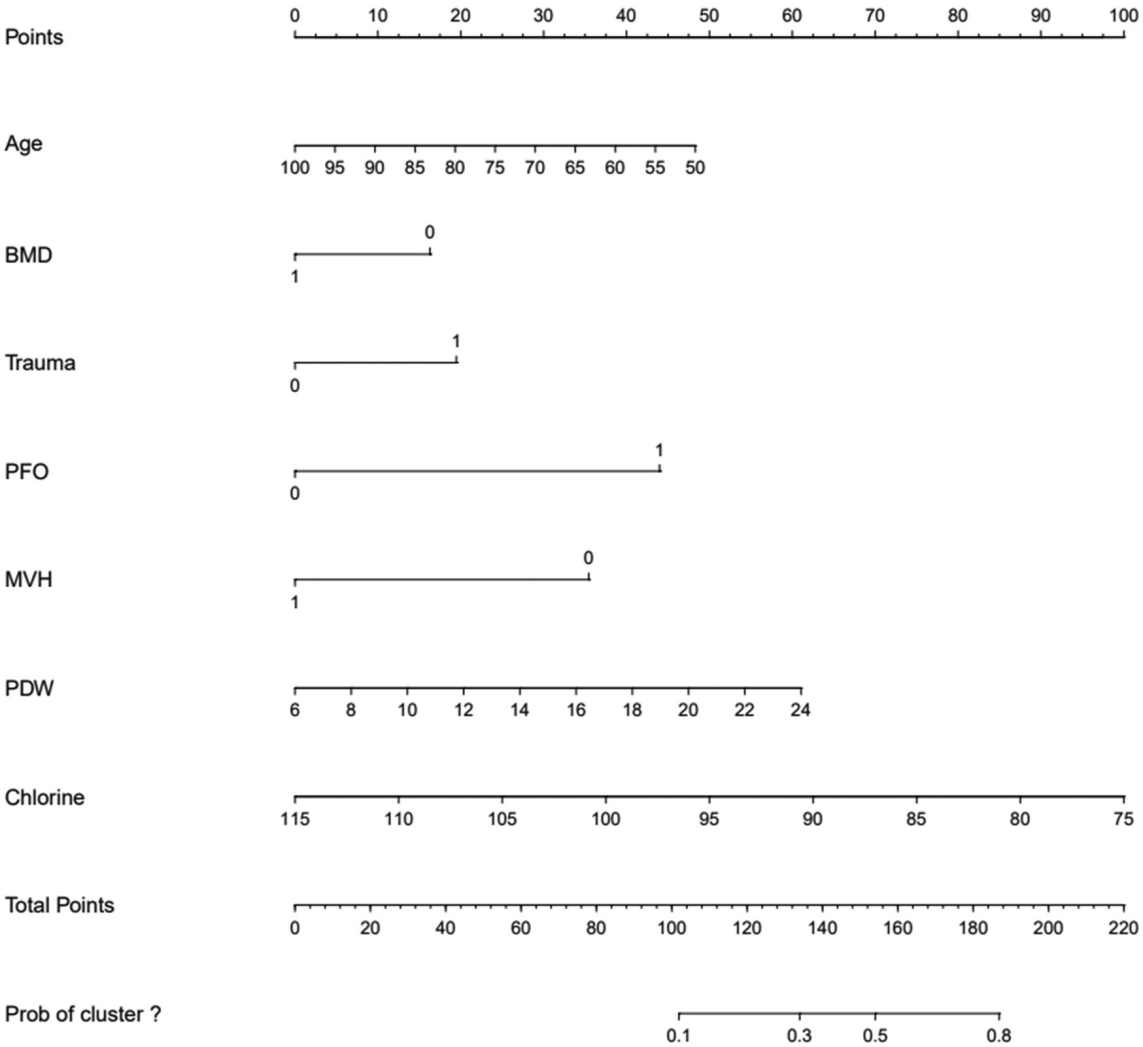
Nomogram for predicting the risk of residual back pain after PKP in OVCF patients.

The ROC curve analysis indicated that the diagnostic value of the combination of independent risk factors was higher than that of each independent risk factor. Therefore, RStudio was used to build a nomogram model to predict the risk of RBP after PKP in patients with OVCF. The total score of the patients is calculated according to the sum of the scores corresponding to the factors in the nomogram model, and the total score was used as a vertical line to intersect the points on the lower risk axis, that is, the risk of RBP after vertebroplasty for osteoporotic vertebral compression fracture (Figure 3, Table 7).

**Table 7:** Nomogram model score for predicting the risk of residual back pain after PKP in OVCF patients.

Variable		Score/point
Age		0.95× (100-Age)
BMD’s |t| >3.5	Yes	0
	No	16.32
Trauma history	Yes	19.58
	No	0
PFO	Yes	44.21
	No	0
PDW		3.47× (PDW-6)
CL		2.55× (115-CL)
MVH restored	Yes	0
	No	35.68

### 3.5 Calibration curve analysis of the model

**Figure 4:**
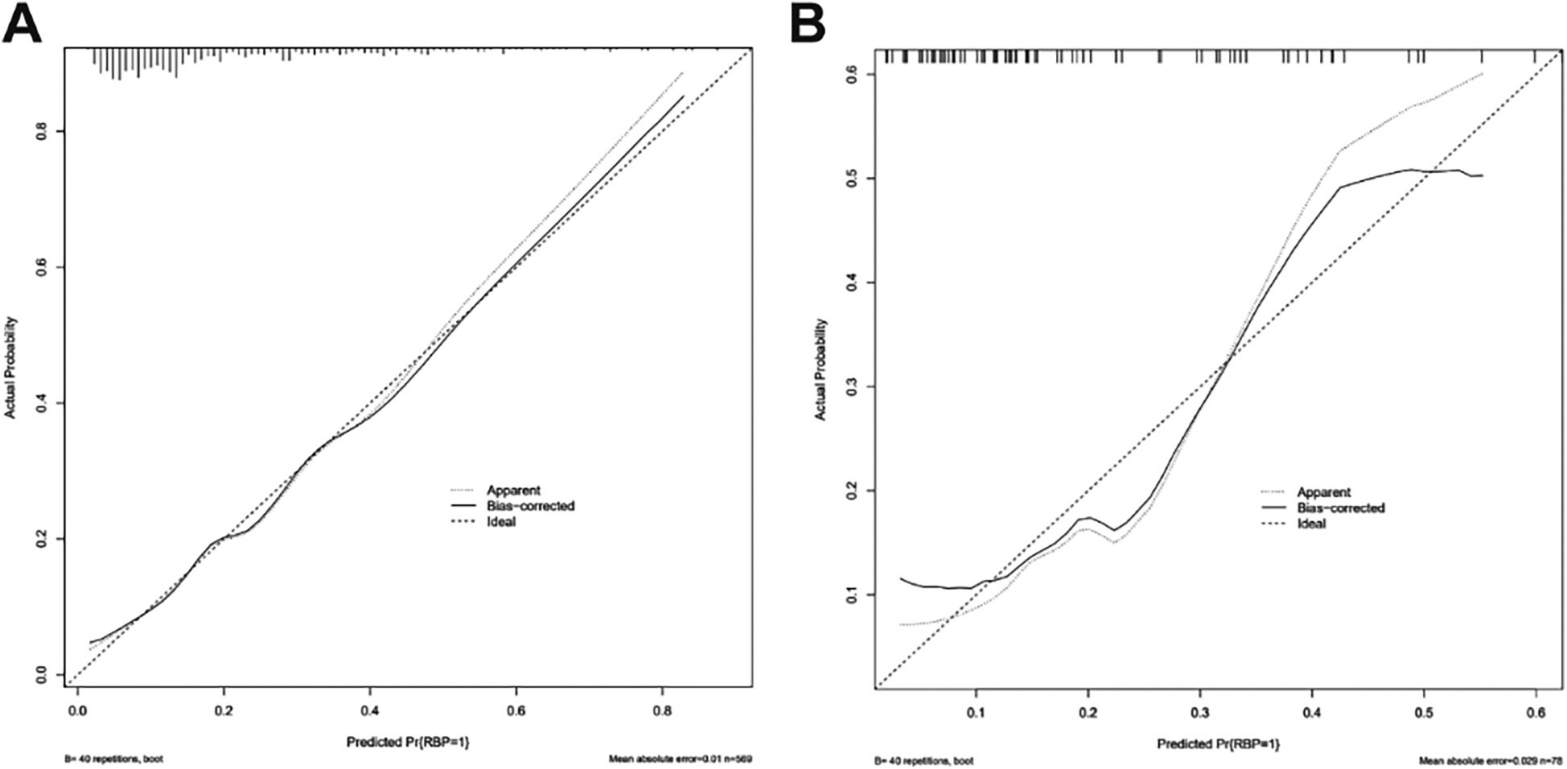
Calibration curve of nomogram model for predicting the risk of residual back pain after PKP in OVCF patients. **(A)** Calibration curve of training set (*n* = 569); **(B)** Calibration curve of validation set (*n* = 78).

The calibration curve of the nomogram was drawn. The standard curve was a straight line passing through the origin of the coordinate axis with a slope of 1. The calibration curves of the model training set and the validation set fell approximately between the standard curve and the acceptable line and fit well with the standard curve. The predicted risk of the nomogram was in good agreement with the actual risk and had a good prediction ability (Figure 4). The Hosmer-Lemeshow test showed that the model training set and the validation set *χ*^2^ were 6.354 and 7.240, respectively. The *P*-values were 0.608 and 0.511, indicating that the model calibration was good.

### 3.6 Internal and external validation of the model

**Figure 5:**
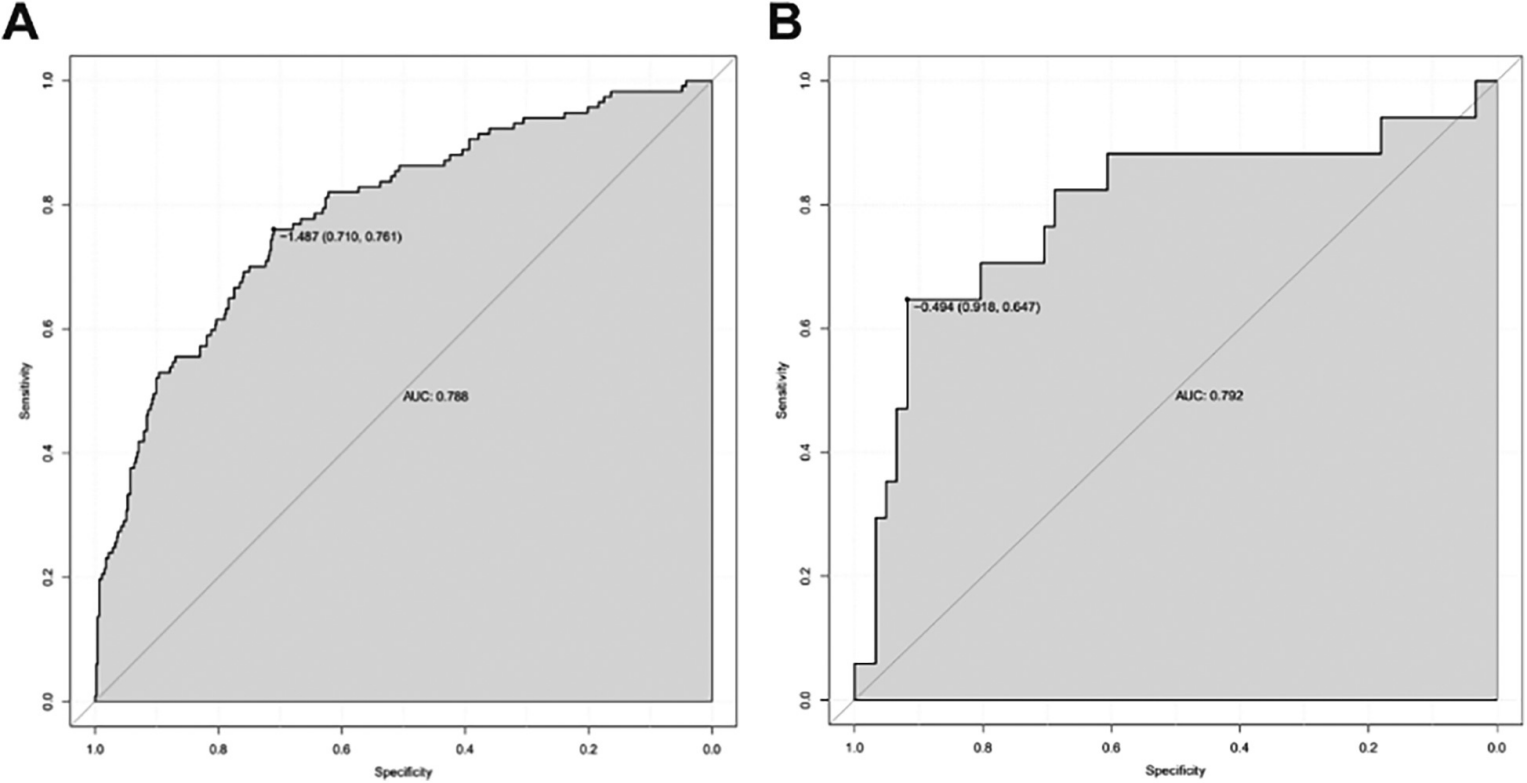
ROC curve obtained using RStudio software. **(A)** ROC curve of the training set (*n* = 569); **(B)** ROC curve of validation set (*n* = 78).

The ROC curve cutoff value of the model training set was 0.184, the specificity and sensitivity were 0.710 and 0.761, respectively, and the AUC was 0.788. Internal validation of the model: after the bootstrap was repeatedly sampled 1,000 times in the training set, the AUC of the model was 0.784. The validation set externally validated the model. The ROC curve cutoff value was 0.379, the specificity and sensitivity were 0.918 and 0.647. The AUC was 0.792, indicating that the model had high discrimination (Figure 5).

### 3.7 Clinical decision curve analysis of model

**Figure 6:**
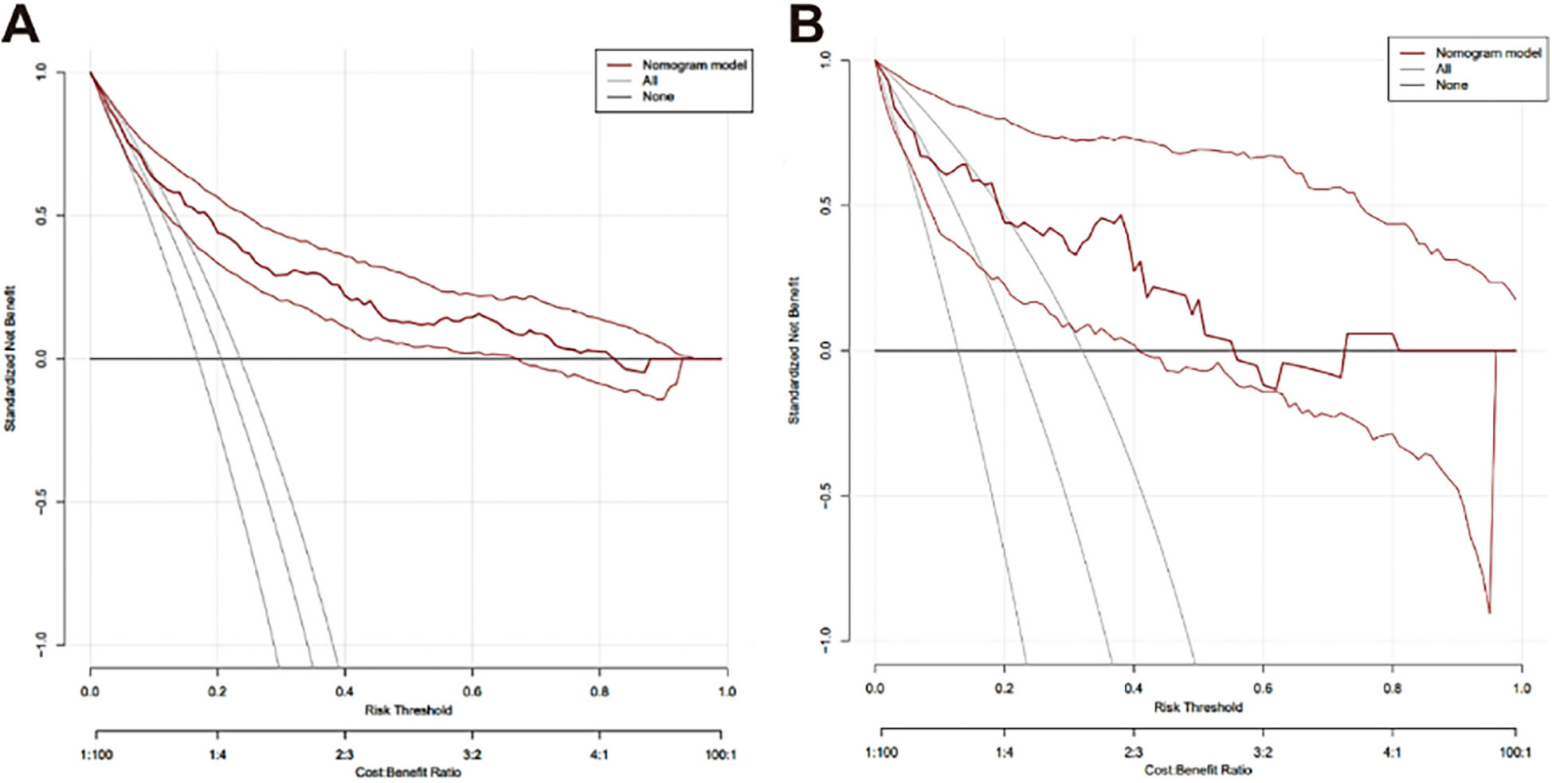
Decision curve analysis for evaluating the net benefit of the nomograph. **(A)** Clinical decision curve of the training set (*n* = 569); **(B)** Clinical decision curve of validation set (*n* = 78).

The clinical decision curve shows that the probability interval of the net benefit threshold of the centralized training model was 6.3%–82.3% and the probability interval of the net benefit threshold of the centralized validation model was 8.7%–55.6%, and 72.5%–81.3%. When the threshold probability of postoperative RBP of patients fell within this interval, the net benefit rate of using this model was significantly higher than that of the “no intervention” and “full intervention” schemes, indicating that the model has good clinical applicability (Figure 6).

## 4 Discussion

The aging population has made OVCF a significant global health issue. Approximately 20% of the world's population is over the age of 70, and at least one in five patients over the age of 50 has one or more vertebral fractures (14, 15). PKP, a modified technique based on PVP, corrects kyphosis caused by vertebral collapse with a balloon. Compared to PVP, PKP contributes to pain relief, restoration of vertebral height, reduction of the average difference in kyphosis wedge angle, and risk of cement leakage (16, 17). Although it has the advantages of small trauma, fast pain relief, early mobilization, and effectively improving the prognosis of patients, some patients still experience postoperative RBP (18). RBP is the most common complication of PKP, with approximately 9%–35% of patients having residual pain after treatment, which is a significant concern for patients (19, 20). However, insufficient attention has been placed on RBP after PKP, with few relevant studies addressing this issue. This study identified several independent risk factors for postoperative RBP in OVCF patients, including young age, high BMD,trauma history, low back fascia edema, high PDW, low serum chloride levels, and no recovery of middle vertebral height.

Previous studies have suggested no statistical difference in the age of patients between the postoperative pain and pain-free groups, indicating that age is not a risk factor for the occurrence of RBP (7). However, this study obtained different results where the younger the age, the higher incidence of RBP in this sample of people over 50 years old. Chen et al. (21) reported that low BMD was an independent risk factor for RBP after PKP. Conversely, the results of this study showed that the incidence of RBP was higher when BMD was 2.5 ≤ |*t*| ≤ 3.5, while patients with preoperative |*t*| > 3.5 were less likely to experience RBP after PKP. For this difference in results, we consider that patients with low BMD have more severe osteoporosis where low energy injuries can cause vertebral compression fractures, while patients with higher BMD have relatively less severe osteoporosis and require greater violence to cause fractures. Thus, patients with higher BMD may have experienced more severe injuries, increasing the likelihood of RBP after PKP.

Moreover, a clear trauma history is also an independent risk factor for RBP after PKP. Osteoporotic vertebral compression fractures usually occur in older adults. Impaired physical coordination, along with a decline in the strength of muscles, ligaments, and other structures, increases their susceptibility to trauma. At the same time, fractures often cause varying degrees of soft tissue damage. The pain from the preoperative fracture may overshadow this, but as the fracture heals, postoperative soft tissue pain becomes more pronounced, leading to RBP. According to Yan et al. (22), the prevalence of fasciopathy in patients with OVCF is as high as 42.1%. Our study found that posterior fascia edema is a strong risk factor (OR = 6.076) for residual pain after PKP. Other studies have also proposed fasciopathy may contribute to residual pain (23, 24). This may be due to the following reasons: (1) lumbar soft tissue injuries, such as superficial fascia and muscle, leads to local pain; (2) pain can be induced by fascial injury and soft tissue edema, which compresses the branches of the dorsal root of the spinal nerve and stimulation of inflammatory factors. PKP is used exclusively for the treatment of vertebral fractures and cannot relieve pain caused by fascia and soft tissue injury. However, it is challenging to determine whether fasciopathy exists preoperatively, with fracture pain masking its presence, or if the pain is a result of intraoperative injury radiating along the sacrospinal muscle.

This study analyzed the results of the preoperative laboratory findings of patients. A high PDW and a low serum chlorine value were independent risk factors for residual pain after PKP. PDW is a parameter that represents the variation of platelet volumes in the blood, which is used to express the degree of homogeneity of platelet. It is also a determinant of platelet activation and is considered a marker of inflammatory disease. During platelet activation, the release of various inflammatory cytokines leads to the formation of platelet clots and the formation of a thrombus (25). A decrease in complement Cl concentrations can enhance the inflammatory response of endothelial cells (26, 27). Cl- reduction is key to foam cell formation and inflammation (28). When the patient was in such a prethrombotic state of inflammatory intensity, an increase in inflammatory factors and poor blood circulation can increase pain. Such pain interferes with early functional exercise activity and reduces muscle strength, leading to further pain. PDW and serum chloride can be used as biomarkers to predict residual pain after PKP in patients with OVCF.

Our study found a statistical difference between the two groups regarding AVH restored and MVH restored, but only the middle height of the vertebral body not recovered was identified as an independent risk factor for the occurrence of residual pain. Denis' three-column theory suggests that the anterior and middle columns of the vertebrae bear 80% of the stress, and the middle column plays a key role in maintaining spinal stability. Balloon dilatation during PKP restores the middle height of the vertebrae, with sufficient cement diffusion, and the chemical and thermal neurolytic effects of PMMA effectively relieve pain at the fracture site (29, 30). The biomechanical environment is restored to equilibrium which brings about an improvement in the compliance of the soft tissues of the back to reduce the possibility of residual pain in the postoperative period (31). Conversely, if the middle vertebral height is not fully restored, the incidence of RBP can be increased. Furthermore, previous studies have shown that facet joint violations, a large number of fracture segments, fracture location, refractive fracture of adjacent or operated vertebral bodies, insufficient dispersion of bone cement in the fracture line area, leakage of bone cement, bone cement volume, inflammatory reaction or local ischemia caused by bone cement, and segmental kyphosis are also independent risk factors for residual pain (19, 32).

The ROC curve shows that the predictive ability of the combined model was stronger than that of each independent factor, indicating that a complete assessment of multiple factors is required to improve accuracy when predicting the risk of RBP. The study established a nomogram that can calculate the risk of RBP in OVCF patients after PKP, which can help to screen high-risk patients and provide a basis for early clinical prevention and treatment. Sample calculations for scores using different variables for an 80-year-old patient can be estimated as follows: patient with OVCF [score 0.95 × (100–80) = 19], lumbar BMD *t* of −2.0 (score 16.32), with a cleartrauma history (score 19.58), posterior fascia edema (score 44.21), and a measured PDW value of 12.80 [3.47 × (12.80–6) = 23.60], and with a serum chlorine value of 105 [score 2.55 × (115–105) = 25.5], if the height of 1/3 of the vertebral body is not recovered (score 35.68), the total score is indicated by the sum of the above scores (183.89) and the corresponding risk value is close to 80%. Therefore, the clinician should pay greater attention to the possibility of RBP after surgery and provide timely intervention.

## 5 Conclusions

This study analyzed the risk factors for RBP after PKP in patients with OVCF. The results showed that young age, high BMD,trauma history, posterior fascia edema, high PDW, low serum chlorine, and no recovery of middle vertebral height were independent risk factors for RBP after PKP in patients with OVCF, and the predictive value of combined factors was higher. Thus, a visual nomogram model was established to predict the risk of residual pain that is simple and easy to read. The nomogram model demonstrated strong predictive ability, good calibration, and high clinical applicability for predicting postoperative RBP in OVCF patients undergoing PKP. The model's practical utility is further supported by its performance in both internal and external validations and decision curve analysis. Therefore, this model can guide clinical risk assessment and take measures against high-risk factors for the timely prevention of RBP.

There are some limitations to this study. Firstly, this study was a retrospective case-control study using a visual analog scale in the medical chart and follow-up records to determine whether a patient experienced RBP, which may be subject to selection bias. Secondly, this study was a single-center study, and the model may not be generalizable to the settings outside the study site. Therefore, prospective studies with large samples, multicenter and long-term follow-up are still needed to verify the validity of the model.

## Data Availability

The original contributions presented in the study are included in the article/Supplementary Material, further inquiries can be directed to the corresponding author/s.

## References

[1] Reid IR McClung MR. Osteopenia: a key target for fracture prevention. Lancet Diabetes Endocrinol. (2024) 12:856–64. 10.1016/S2213-8587(24)00225-0 39326428

[2] Si L Winzenberg TM Jiang Q Chen M Palmer AJ. Projection of osteoporosis-related fractures and costs in China: 2010–2050. Osteoporos Int. (2015) 26:1929–37. 10.1007/s00198-015-3093-2 25761729

[3] Wen Z Mo X Zhao S Qi Z Fu D Wen S Study on risk factors of primary non-traumatic OVCF in Chinese elderly and a novel prediction model. Orthop Surg. (2022) 14:2925–38. 10.1111/os.13531 36168985 PMC9627056

[4] Parreira PCS Maher CG Megale RZ March L Ferreira ML. An overview of clinical guidelines for the management of vertebral compression fracture: a systematic review. Spine J. (2017) 17:1932–8. 10.1016/j.spinee.2017.07.174 28739478

[5] Wang W Liu Y Wan H Zeng L Peng Z Yang D Effectiveness and prognostic factors of different minimally invasive surgeries for vertebral compression fractures. BMC Musculoskelet Disord. (2023) 24:11. 10.1186/s12891-022-06125-8 36609293 PMC9817397

[6] Ma Y Wu X Xiao X Ma Y Feng L Yan W Effects of teriparatide versus percutaneous vertebroplasty on pain relief, quality of life and cost-effectiveness in postmenopausal females with acute osteoporotic vertebral compression fracture: a prospective cohort study. Bone. (2020) 131:115154. 10.1016/j.bone.2019.115154 31733423

[7] Lin M Wen X Huang Z Huang W Zhang H Huang X A nomogram for predicting residual low back pain after percutaneous kyphoplasty in osteoporotic vertebral compression fractures. Osteoporos Int. (2023) 34:749–62. 10.1007/s00198-023-06681-2 36738335

[8] Yang JS Liu JJ Chu L Li J Chen C Chen H Causes of residual back pain at early stage after percutaneous vertebroplasty: a retrospective analysis of 1,316 cases. Pain Physician. (2019) 22:E495–e503. 31561662

[9] Luo Y Jiang T Guo H Lv F Hu Y Zhang L. Osteoporotic vertebral compression fracture accompanied with thoracolumbar fascial injury: risk factors and the association with residual pain after percutaneous vertebroplasty. BMC Musculoskelet Disord. (2022) 23:343. 10.1186/s12891-022-05308-7 35410277 PMC8996573

[10] Xia W Fu H Zhu Z Liu C Wang K Xu S Association between back muscle degeneration and spinal-pelvic parameters in patients with degenerative spinal kyphosis. BMC Musculoskelet Disord. (2019) 20:454. 10.1186/s12891-019-2837-0 31630684 PMC6802345

[11] Li Q Shi L Wang Y Guan T Jiang X Guo D A nomogram for predicting the residual back pain after percutaneous vertebroplasty for osteoporotic vertebral compression fractures. Pain Res Manag. (2021) 2021:3624614. 10.1155/2021/3624614 34760032 PMC8575618

[12] Bo J Zhao X Hua Z Li J Qi X Shen Y. Impact of sarcopenia and sagittal parameters on the residual back pain after percutaneous vertebroplasty in patients with osteoporotic vertebral compression fracture. J Orthop Surg Res. (2022) 17:111. 10.1186/s13018-022-03009-4 35184761 PMC8859872

[13] Skjødt MK Abrahamsen B. New insights in the pathophysiology, epidemiology, and response to treatment of osteoporotic vertebral fractures. J Clin Endocrinol Metab. (2023) 108:e1175–85. 10.1210/clinem/dgad256 37186550

[14] Karmakar A Acharya S Biswas D Sau A. Evaluation of percutaneous vertebroplasty for management of symptomatic osteoporotic compression fracture. J Clin Diagn Res. (2017) 11:Rc07–rc10. 10.7860/JCDR/2017/25886.10461 28969223 PMC5620864

[15] Kendler DL Bauer DC Davison KS Dian L Hanley DA Harris ST Vertebral fractures: clinical importance and management. Am J Med. (2016) 129:221.e1–10. 10.1016/j.amjmed.2015.09.020 26524708

[16] Hoyt D Urits I Orhurhu V Orhurhu MS Callan J Powell J Current concepts in the management of vertebral compression fractures. Curr Pain Headache Rep. (2020) 24:16. 10.1007/s11916-020-00849-9 32198571

[17] Wang B Zhao CP Song LX Zhu L. Balloon kyphoplasty versus percutaneous vertebroplasty for osteoporotic vertebral compression fracture: a meta-analysis and systematic review. J Orthop Surg Res. (2018) 13:264. 10.1186/s13018-018-0952-5 30348192 PMC6196425

[18] Patel D Liu J Ebraheim NA. Managements of osteoporotic vertebral compression fractures: a narrative review. World J Orthop. (2022) 13:564–73. 10.5312/wjo.v13.i6.564 35949707 PMC9244957

[19] Li Y Yue J Huang M Lin J Huang C Chen J Risk factors for postoperative residual back pain after percutaneous kyphoplasty for osteoporotic vertebral compression fractures. Eur Spine J. (2020) 29:2568–75. 10.1007/s00586-020-06493-6 32507918

[20] Xu JJ Tang XT Yang J Wang YH Zhu DC Wu YS The effect of medial branch block on postoperative residual pain relieve after percutaneous kyphoplasty: a randomized controlled trial with 12-month follow-up. Pain Physician. (2021) 24:E1059–e1066. 34704715

[21] Chen C Wu B Yu H Dai Z Yan L Cai D Association between vertebral bone quality score and residual back pain following percutaneous vertebroplasty for osteoporotic vertebral compression fractures. Eur Spine J. (2025) 34(2):537–45. 10.1007/s00586-024-08619-6 39688705

[22] Yan Y Xu R Zou T. Is thoracolumbar fascia injury the cause of residual back pain after percutaneous vertebroplasty? A prospective cohort study. Osteoporos Int. (2015) 26:1119–24. 10.1007/s00198-014-2972-2 25510580

[23] Yang XG Dong YQ Liu X Liu XL Luo HT Bao Y Incidence and prognostic factors of residual back pain in patients treated for osteoporotic vertebral compression fractures: a systematic review and meta-analysis. Eur Spine J. (2024) 33:4521–37. 10.1007/s00586-024-08426-z 39103616

[24] Ge C Chen Z Lin Y Zheng Y Cao P Chen X. Preoperative prediction of residual back pain after vertebral augmentation for osteoporotic vertebral compression fractures: initial application of a radiomics score based nomogram. Front Endocrinol. (2022) 13:1093508. 10.3389/fendo.2022.1093508 PMC9816386 36619583

[25] Kaiser R Escaig R Nicolai L. Hemostasis without clot formation: how platelets guard the vasculature in inflammation, infection, and malignancy. Blood. (2023) 142:1413–25. 10.1182/blood.2023020535 37683182

[26] Valdivieso ÁG Santa-Coloma TA. The chloride anion as a signalling effector. Biol Rev Camb Philos Soc. (2019) 94:1839–56. 10.1111/brv.12536 31231963

[27] Yang H Huang LY Zeng DY Huang EW Liang SJ Tang YB Decrease of intracellular chloride concentration promotes endothelial cell inflammation by activating nuclear factor-*κ*B pathway. Hypertension. (2012) 60:1287–93. 10.1161/HYPERTENSIONAHA.112.198648 23006728

[28] Wu QQ Liu XY Xiong LX Shang JY Mai XY Pang RP Reduction of intracellular chloride concentration promotes foam cell formation. Circ J. (2016) 80:1024–33. 10.1253/circj.CJ-15-1209 26911455

[29] Zhu J Yang S Cai K Wang S Qiu Z Huang J Bioactive poly (methyl methacrylate) bone cement for the treatment of osteoporotic vertebral compression fractures. Theranostics. (2020) 10:6544–60. 10.7150/thno.44428 32483469 PMC7255031

[30] Seesala VS Sheikh L Basu B Mukherjee S. Mechanical and bioactive properties of PMMA bone cement: a review. ACS Biomater Sci Eng. (2024) 10:5939–59. 10.1021/acsbiomaterials.4c00779 39240690

[31] Mei X Sun ZY Zhou F Luo ZP Yang HL. Analysis of Pre- and postoperative pain variation in osteoporotic vertebral compression fracture patients undergoing kyphoplasty. Med Sci Monit. (2017) 23:5994–6000. 10.12659/MSM.906456 29252980 PMC5743174

[32] Yang D Liu X Zhou Y Xu Y Huang Q. A novel scoring system to predict the residual back pain after percutaneous kyphoplasty for osteoporotic vertebral compression fracture. Front Surg. (2022) 9:1035681. 10.3389/fsurg.2022.1035681 36311951 PMC9606611

